# Successful Embolectomy of the Plantar Artery Occlusion Due to Thromboembolism

**DOI:** 10.3400/avd.cr.20-00125

**Published:** 2020-12-25

**Authors:** Kazunori Hashimoto, Harunobu Matsumoto, Takao Nonaka, Naoyuki Kimura, Koichi Yuri, Atsushi Yamaguchi

**Affiliations:** 1 Department of Cardiovascular Surgery, Jichi Medical University Saitama Medical Center, Saitama, Saitama, Japan

**Keywords:** thromboembolism, limb ischemia, abdominal aorta

## Abstract

A 76-year-old man was admitted to our hospital because of sudden pain in the left leg. Computed tomography and ultrasonography findings revealed occlusion of the plantar and sural arteries and atherothrombosis in the abdominal aorta, and thromboembolism was suspected. The foot was treated for ischemia and embolic sources in two stages. First, we performed embolectomy using a balloon catheter exposed to the common plantar artery through arteriotomy. This surgical revascularization is an effective treatment method for thromboembolism. Four weeks later, we performed graft replacement of the abdominal aorta to prevent thromboembolism.

## Introduction

Tibial and pedal artery embolism is rarely encountered, and most emboli that cause peripheral arterial disease are large and often occlude the proximal arteries. Retrograde embolectomy of tibial emboli through the pedal arteries has been reported previously.^1)^ However, reports of surgical revascularization for pedal arterial occlusion due to peripheral embolism are rare. One important cause of peripheral embolism may be an aortic thrombus. Arterial thromboembolism in patients with an unknown source of embolization is still associated with significant morbidity and mortality. Furthermore, spontaneous embolization from an aortic plaque can lead to organ dysfunction and limb loss.^2)^ Atherosclerosis of the abdominal aorta is considered a significant risk factor for embolism. A computed tomography (CT) angiography provides information on characteristics of the aortic wall and enables evaluation of aortic plaques that could form emboli.^3)^ We report here the successful embolectomy of the plantar artery explored at ankle level and prevention of embolization recurrence with a graft replacement of the abdominal aorta, which was confirmed as the source of embolization.

## Case Report

**Figure 1:**
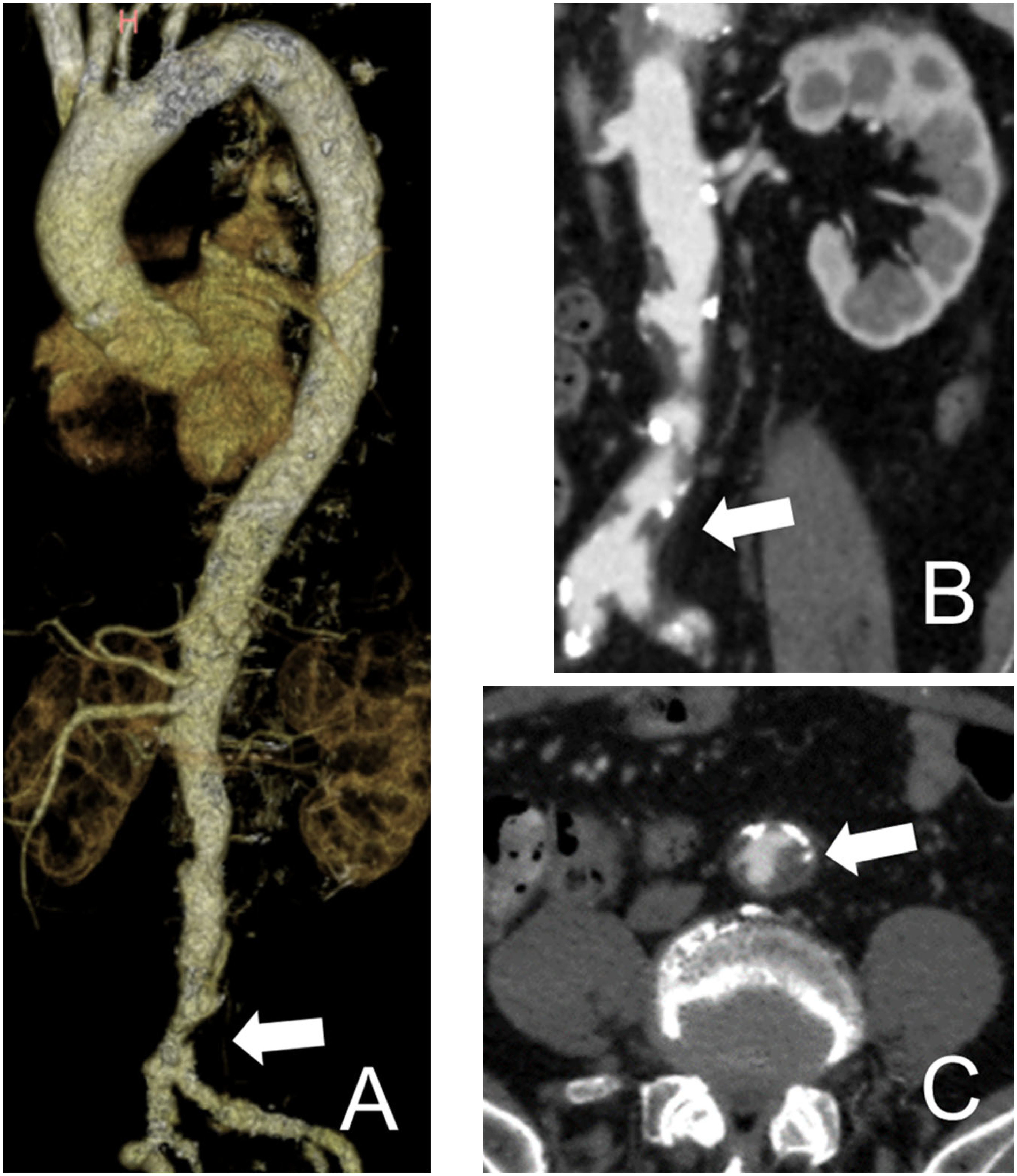
Fig. 1 (**A**) Three-dimensional computed tomography (CT) image. (**B**) Multiplanar reconstruction image. (**C**) Cross-sectional image of a preoperative enhanced CT scan. The atherothrombosis is floating in the abdominal aorta.

**Figure 2:**
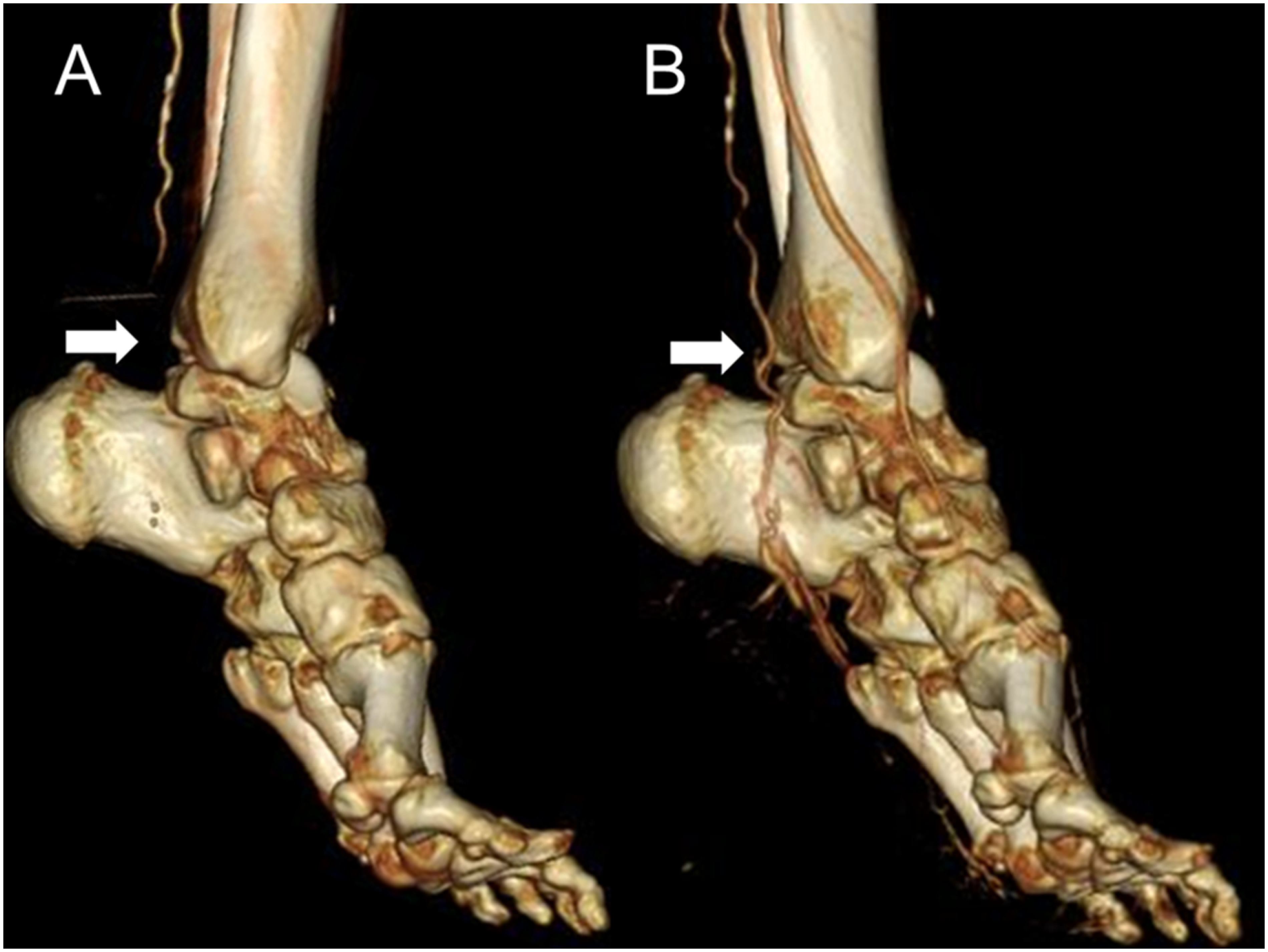
Fig. 2 (**A**) Preoperative computed tomography (CT) angiography showing a patent posterior tibial artery. The plantar artery and the dorsal artery at the level of the ankle are occluded. (**B**) Postoperative CT angiography showing that the common plantar artery is patent.

A 76-year-old man was admitted to our hospital with the complaint of acute pain in the left lower limb and a pale foot. His medical history included hypertension, hyperlipidemia, and cerebral infarction. Arterial pulsation was palpable in the posterior tibial artery of the left leg, but it was unpalpable in the dorsalis pedis artery. The ankle–brachial pressure index of the left leg was within the normal range. The pulse of the left foot was recognized, and the patient maintained left foot motor function; however, the forepart of the left foot had ischemia and perceptual dysfunction. The ultrasonography findings revealed that the posterior tibial artery was patent, but the plantar artery was occluded. The presence of local occlusive lesions was suspected. The diameter of the common plantar artery was 3.0 mm, and its echo brightness was slightly high. A slight color Doppler was found on the margin of the plantar artery lumen. Fresh thrombosis or a plaque was suspected in the lumen of the common plantar artery. A CT scan revealed insufficient arterial circulation in the sural muscle of the left lower limb. The posteior tibial artery above the patient’s ankle was patent, but the plantar artery was occluded in addition to the anterior tibial artery and the dorsalis pedis artery. There was prominent atherosclerosis in the wall of the abdominal aorta. In the lumen of the abdominal aorta, atherothrombosis causing embolism was found. Compared to the abdominal aorta, no thoracic aorta or thoracic abdominal aorta was found to be likely to cause embolism (**Figs. 1** and **2A**).

We decided to perform an emergency operation to treat the lower limb ischemia. We incised the common plantar artery directly and removed the thrombus using a balloon catheter under general anesthesia. The left ankle was incised linearly, and along with the common plantar artery, the lateral and medial plantar arteries were exposed and taped. Following heparinization, blood flow to the common plantar artery was blocked. After transversely incising the common plantar artery, proximal and distal embolectomy was performed using a 2 Fr balloon catheter, directed proximally to the posterior tibial artery and distally to the lateral and medial plantar arteries. After the thrombus was removed, sufficient backflow through the distal side of the common plantar artery was confirmed. The arteriotomy was closed with an interrupted 7-0 monofilament polypropylene suture.

Heparin was used until the next day as anticoagulant therapy after embolectomy. Although the dorsalis pedis artery was occluded, the Doppler sound of the plantar artery region became audible; therefore, we decided that no further revascularization was necessary.

**Figure 3:**
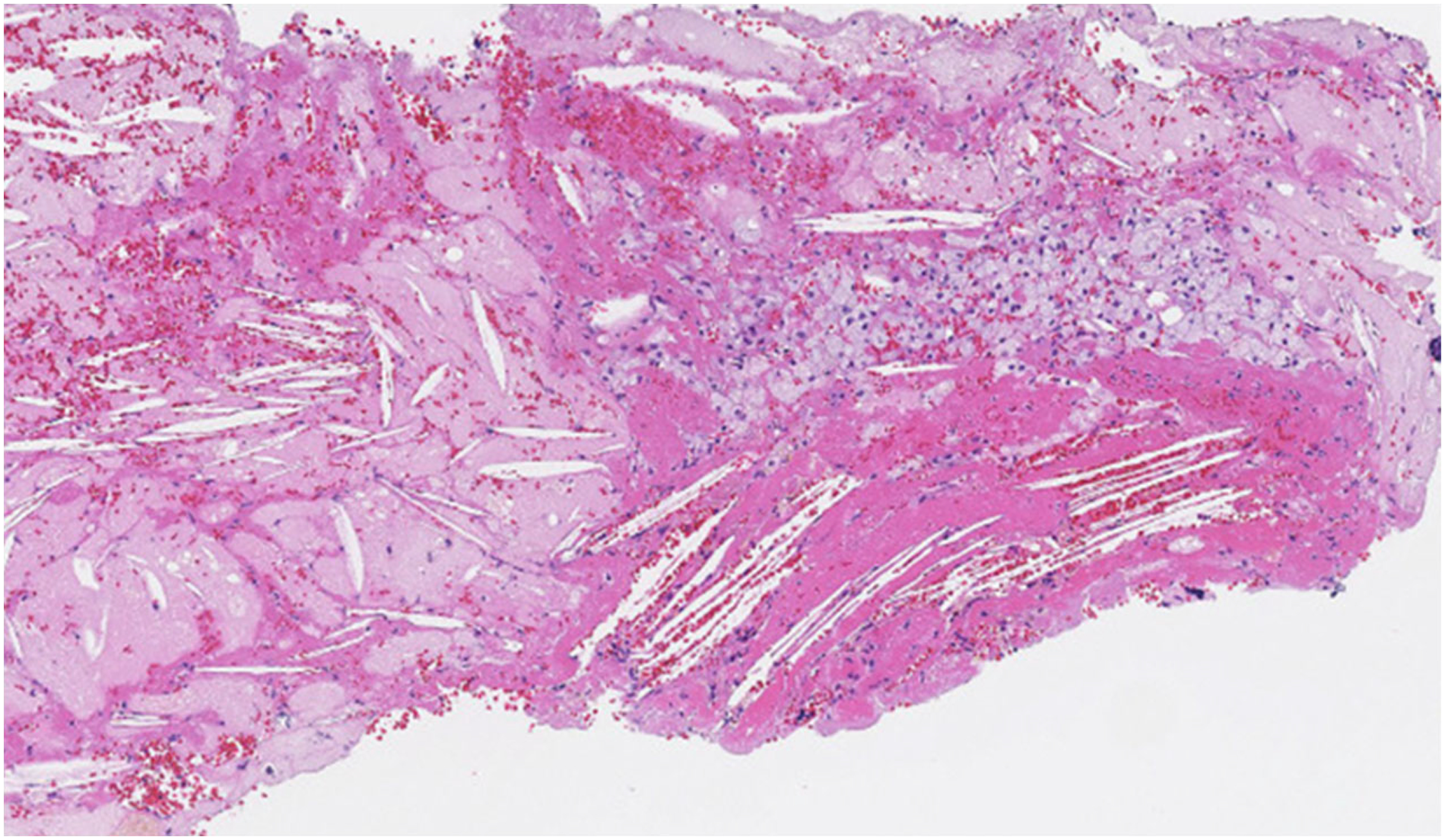
Fig. 3 Hematoxylin and eosin staining at low magnification showed cholesterol crystals in atherothrombosis.

After examining the thrombosis, the pathological diagnosis was a cholesterol crystal (**Fig. 3**). The circulation of his left foot and pain of the left calf improved postoperatively, and postoperative CT showed a patent plantar artery (**Fig. 2B**).

The patient was discharged from the hospital after the numbness and pain in his right leg improved. We ruled out the possibility of the abdominal aorta as the origin of embolism. Hence, the patient was hospitalized again and underwent a graft replacement of the abdominal aorta. During the 4 weeks between the day of the embolectomy and the graft replacement, he had taken time to rehabilitate because of pain in the gastrocnemius muscle and had undergone preoperative tests, including an evaluation of cardiac function. His postoperative course was uneventful, and no recurrence was observed 1 year postoperatively.

## Discussion

Primary thromboembolic occlusion of the pedal vessels is rare. A thrombus or plaque embolization resulting from an aneurysm or atherosclerotic lesion has been reported to cause a peripheral embolism.^4)^ Standard treatment of reversible ischemia from an arterial embolism involves immediate heparinization, resuscitation, stabilization of the patient’s condition, embolectomy, and treatment of the embolic source. Thus, the Fogarty arterial embolectomy catheter is of major importance in this treatment.^5)^ By contrast, a recent report indicated that endovascular treatment is useful for mechanical thrombus suction devices.^6,7)^ An endovascular intervention for acute limb ischemia includes catheter-directed thrombolysis (CDT) and a percutaneous aspiration thrombectomy.

Although CDT is effective, it can increase the risk of major bleeding and stroke. Moreover, CDT has no effect on hard embolic debris.^6)^ In particular, thromboembolism has been reported to develop following a catheter-guided procedure.^2)^ Furthermore, surgical transfemoral embolectomy below the knee may be ineffective as it is difficult to guide a catheter to crural and foot arteries compared to thrombus aspiration therapy.

One solution to the problems of distal embolization and an incomplete transfemoral approach is direct exposure at tibial and ankle arteries. The surgical technique of embolectomy has been attempted for the ankle,^1,5)^ with a reported salvage rate of 79%.^5)^ Thrombus confined to the infrapopliteal arteries is a major indication for this procedure.

Based on the ultrasonography findings in this case that indicated that “fresh thrombosis or plaque was suspected” in the pedal vessels, we chose embolectomy because it was more reliable than endovascular intervention. A diagnosis based on ultrasonography findings can be used for the morphological evaluation of thrombus in plantar arteries, providing indications for surgery.^8)^ In this case, the common plantar artery, anterior tibial artery, and dorsalis pedis artery were occluded. However, based on the findings of the color Doppler entering on the margin of the plantar artery, we determined that the thrombus in the plantar artery was relatively fresh and could be removed. Since the diameter of the plantar artery was relatively large at 3 mm, we decided that the procedure would be easy. A transverse incision was made in the plantar artery, and the incision was repaired with an interrupted suture. A thinner plantar artery would require a longitudinal incision, and its repair would require a vein patch.

The presence of a mobile thrombus in the aorta is an uncommon pathology with drastic embolic complications. Recurrent spontaneous distal emboli may result from thrombi in the abdominal aortic ulcers.^3)^ The infrarenal aorta is the most common source of emboli. The source of the embolus must be removed to prevent the recurrence of embolism, and in our case, a mobile thrombus was detected in the abdominal aorta using CT and echo findings.

CT angiography is the preferred modality for planning surgeries for a known or suspected atherosclerotic aorta.^3)^ Treatments to prevent recurrence of embolization include antithrombotic and anticoagulant therapy, endarterectomy, surgical replacement, or exclusion; however, an optimal treatment has not been established. A high recurrence rate of 75% has been reported for patients who receive medical treatment only, especially anticoagulant therapy alone.^9)^

The most common surgical approaches for eliminating the source of the embolus are thromboendarterectomy or resection and graft replacement.^2)^ If the source of embolism is extensive atherosclerosis that extends to the suprarenal abdominal aorta or the descending thoracic aorta, there is a higher risk of patient mortality.^9)^ Extra-anatomical arterial reconstruction with exclusion of the proximal source and endovascular exclusion has been recommended for patients at high risk.^2,4)^

Surgical approaches have a perioperative mortality rate of 2.6% to 20%. The rate of recurrence of post-surgery emboli is 5%–25%, and the rates of toe and leg amputations are 10% and 11%, respectively. Although less invasive treatments of endovascular exclusion have been reported, the challenges associated with intraoperative embolism due to catheter manipulation, as well as their long-term results, are unknown.^4,9)^ Surgical treatment is recommended in patients with a low risk of mortality and morbidity if the embolic source is identified and accessible.^9)^ In the present case, the patient’s activity level before his original hospital visit was high, and organ damage was not observed; therefore, the operative risk was low. One way to perform safe surgery is to prevent intraoperative embolization. As for the clamping placement, distal clamping before proximal clamping is required to avoid lesions with an irregular plaque and a thrombus.

In this case, we determined that the abdominal aorta was the source of the embolus as pathological examination indicated that the thrombus removed from the plantar artery contained cholesterol crystals. To our knowledge, no reports have suggested whether graft replacement or thromboendarterectomy is better. We determined that graft replacement could be a reliable surgical approach for eliminating the infrarenal aorta of the embolus because this approach is more familiar to us than thromboendarterectomy.

## Conclusion

We report the successful embolectomy for occlusion of the plantar artery and the prevention of embolization recurrence with a graft replacement of the abdominal aorta, confirmed as the embolization source. These surgical techniques can prevent the risk of recurrence of embolism and amputation of the lower extremities.

## References

[1] Hardy R, Garnham A, Samman Y, et al. Microtibial embolectomy. Eur J Vasc Endovasc Surg 2003; 25: 35-9. 12525809 10.1053/ejvs.2002.1768

[2] Dougherty MJ, Calligaro KD. Endovascular treatment of embolization of aortic plaque with covered stents. J Vasc Surg 2002; 36: 727-31. 12368733

[3] Reber PU, Patel AG, Stauffer E, et al. Mural aortic thrombi: an important cause of peripheral embolization. J Vasc Surg 1999; 30: 1084-9. 10587393 10.1016/s0741-5214(99)70047-9

[4] Zhang WW, Abou-Zamzam AM, Hashisho M, et al. Staged endovascular stent grafts for concurrent mobile/ulcerated thrombi of thoracic and abdominal aorta causing recurrent spontaneous distal embolization. J Vasc Surg 2008; 47: 193-6. 18178473 10.1016/j.jvs.2007.07.050

[5] Youkey JR, Clagett GP, Cabellon S Jr, et al. Thromboembolectomy of arteries explored at the ankle. Ann Surg 1984; 199: 367-71. 6703798 10.1097/00000658-198403000-00020 PMC1353407

[6] Cho S, Lee SH, Joh JH. Complete revascularization of acute limb ischemia with distal pedal access. Vasc Endovascular Surg 2020; 54: 69-74. 31500525 10.1177/1538574419873177

[7] Vorwerk D, Triebe S, Ziegler S, et al. Percutaneous mechanical thromboembolectomy in acute lower limb ischemia. Cardiovasc Intervent Radiol 2019; 42: 178-85. 30488304 10.1007/s00270-018-2129-3

[8] Nicholls SC, Smith W. Peripheral arterial embolization: Doppler ultrasound scan diagnosis. J Vasc Surg 2000; 31: 811-4. 10753292 10.1067/mva.2000.102324

[9] Keen RR, McCarthy WJ, Shireman PK, et al. Surgical management of atheroembolization. J Vasc Surg 1995; 21: 773-81; discussion, 780-1. 7769735 10.1016/s0741-5214(05)80008-4

